# Supporting Women’s Participation in Developing A Seaweed Supply Chain in Kiribati for Health and Nutrition

**DOI:** 10.3390/foods9040382

**Published:** 2020-03-26

**Authors:** Libby Swanepoel, Tereere Tioti, Taati Eria, Karibanang Tamuera, Ulusapeti Tiitii, Silva Larson, Nicholas Paul

**Affiliations:** 1 School of Health and Sport Sciences, University of the Sunshine Coast, Maroochydore 4558, Queensland, Australia; 2 Ministry of Fisheries and Marine Resource Development, PO Box 64, Bairiki, Tarawa, Kiribati; ttioti26@gmail.com (T.T.); taatie@fisheries.gov.ki (T.E.); karibanangt@mfmrd.gov.ki (K.T.); 3 Ministry of Agriculture and Fisheries, Apia WS1300, Samoa; Sapeti.Tiitii@maf.gov.ws; 4 School of Science and Engineering, University of the Sunshine Coast, Maroochydore 4558, Queensland, Australia; silva.larson@gmail.com (S.L.); npaul@usc.edu.au (N.P.)

**Keywords:** seaweed, *Kappaphycus*, *Caulerpa*, *Acanthophora*, carrageenan, sea grapes, peer-led training, sustainable diets, Pacific, SDGs

## Abstract

Seaweeds are a source of food throughout the Pacific region. Kiribati, however, does not have a strong history of using seaweed in their diets, despite having reliable access to indigenous edible seaweeds. A series of peer-led seaweed training workshops held in Kiribati between 2018 and 2019 provided women with knowledge, skills, and motivational support needed to engage in the seaweed supply chain, from harvesting, processing, and marketing to consumption. This study aimed to identify opportunities and enablers to support women’s participation across the seaweed supply chain. Structured interviews with 49 women explored their interest and expected costs and benefits from involvement in the supply chain. There was high interest in most seaweed-related activities and the key motivators were health and nutrition for themselves and their family. Participants were also interested in developing and sharing new skills and saw the potential for income generation. However, there were also clear barriers including a desire for further training in seaweed harvesting, processing, and recipe creation; additional social support; and in public promotion. Given the natural resources and desire of women to engage in developing this new edible seaweed supply chain in Kiribati, there is now a need for capacity development to build social and economic wellbeing and food security across the broader community. Additional peer-to-peer training opportunities may look to other Pacific Islands where seaweed is already an established and traditional food.

## 1. Introduction

Pacific Island nations face considerable challenges including urbanisation, climate change [1], and some of the highest rates of malnutrition in the world [2]. Changes in dietary patterns in the Pacific have resulted in a triple burden of malnutrition, with growth in diet-related non-communicable diseases alongside micronutrient deficiencies, stunting, and wasting [2,3,4]. Transition to a modern diet has seen an increase in nutrient-poor, energy-dense foods, leading to stunted growth together with weight gain, resulting in poor health outcomes throughout the life-course [2,5]. Competitive pricing of non-traditional processed foods has additionally suppressed the production and consumption of local food products in the Pacific [6], including edible seaweeds.

Located in the Micronesian region of the Pacific Ocean, the Republic of Kiribati is an archipelago nation consisting of 33 coral atolls with a population of approximately 120,000 people [7]. Ranked as the poorest country in the Pacific Region and 25th poorest internationally [8], Kiribati has experienced a modern nutrition transition and is impacted by sustainable development issues, concerns for food and nutrition security, and the triple burden of malnutrition [9,10,11]. Given its isolated geographical location, Kiribati is particularly vulnerable to the health impacts of globalisation. A consistent rise in the prevalence of adult obesity (currently estimated to be 45.6%), together with an increase in anaemia among women of reproductive age (26.1%) are some of the key health challenges in Kiribati [7].

Food intake is a key factor associated with the rise of non-communicable diseases, with one in five deaths globally associated with poor diets [12]. Issues of malnutrition in Kiribati [10], and elsewhere in the Pacific [13], are attributed to a monotonous diet with poor diversity of foods. Economics, accessibility, knowledge, skills, cultural and social aspects, and awareness for a need to change are important determinants when promoting dietary change [14]. Seaweeds are local and traditional food resources that are highly nutritious, low in cost, easy to harvest or grow, and used extensively in other countries including Samoa, Fiji, and Indonesia [15,16]. Kiribati, however, does not have a strong history of using seaweed in their diet, despite having reliable access to sustainable seaweed gardens on the surrounding reefs.

Seaweeds are consumed globally and while the nutrient composition varies with genus, geography, and season, they are often rich in vitamins A, B, C, and E, various minerals, fibre, and in some cases, protein [17,18]. In addition to an important food crop, seaweed is a valuable small-scale aquaculture practice in the Pacific Islands, particularly the red seaweed *Kappaphycus alvarezii* that is farmed for the gel carrageenan. The social closeness of short food supply chains (such as seaweed) emphasise growth in social and cultural capital, and territorial cohesion [19]. Seaweed farming generates cash income for individuals, families, and villages, often with minimal impact on the environment [20]. Despite this, women’s work in small-scale aquaculture (including seaweed farming) is frequently unrecognised, under-, or unpaid [20]. Social sustainability of seaweed food supply chains must consider fairness among all actors [19]. Active support and engagement of women in developing new seaweed-based food chains in Kiribati is needed to build social and economic wellbeing and food and nutrition security across the broader community. Through a series of peer-led training workshops focused on women’s social, economic, and nutritional wellbeing, this study introduced i-Kiribati women to various activities across the seaweed supply chain including shallow reef harvesting, processing, cooking, consumption, and marketing. To understand how to support women’s ongoing engagement in seaweed activities, this study aimed to determine their interest in involvement and to explore barriers and enablers for participation across the food supply chain.

## 2. Materials and Methods

### 2.1. Location

**Figure 1:**
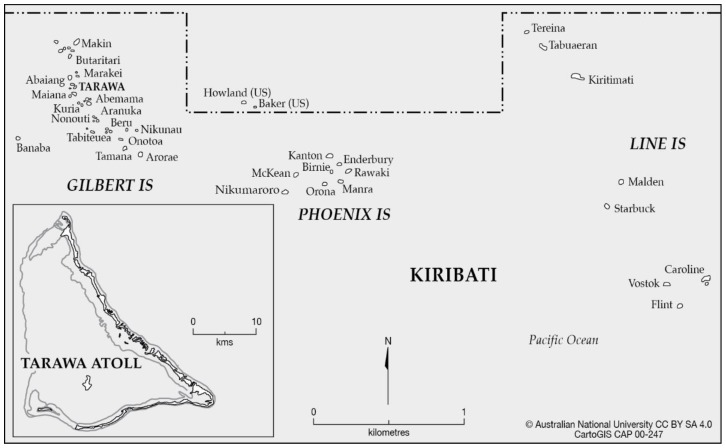
Map of Kiribati in the central Pacific Ocean, with Tarawa Atoll inset reproduced from the Australian National University [22].

This study was undertaken in South Tarawa, the capital of Kiribati and home to almost half (60.000 people) of Kiribati’s total population [21], and most government, commercial, and education facilities (Figure 1).

### 2.2. Training Workshops

Two three-day seaweed training workshops were held in South Tarawa in 2018 and 2019. The first workshop focused on wild harvest and processing of sea grapes (multiple *Caulerpa* species—C. *racemosa* and *C. chemnitzia* [23]) and took a peer-to-peer cross-country training approach that brought Samoan seaweed farmers and Fisheries officers with expertise in sea grape harvesting and processing to Tarawa to train local women. The second workshop focused on nutrition, co-creation of recipes, and practical cooking with three edible seaweeds indigenous to Kiribati, *Caulerpa*, *Kappaphycus*, and *Acanthophora* species (Table 1). Workshop design was based on place-informed education to ensure learning activities incorporated an understanding of the local environment, culture, and fisheries knowledge [24]. Low-literacy learning underpinned all activities, providing opportunities for participants to learn through visual, experiential, practical, and discussion methods [24]. The Kiribati Women’s Council invited women who were active members of recognised community groups, representing all 13 villages in Tarawa to attend workshops. Workshop participants were provided with free transport to the workshop venue and meals to facilitate involvement.

### 2.3. Recruitment of Participants

Structured interviews were conducted at the conclusion of each workshop in June 2018 and July 2019. Participants were voluntarily recruited by the lead author and the locally trained enumerators. Eligibility criteria included adult residents (>18 years) attending either of the seaweed training workshops. Involvement in the structured interview was voluntary and participation had no impact on participant’s involvement in the workshops, however, all the workshop participants opted to also participate in the interview. A research project information sheet was available for participants and they all gave informed consent for inclusion before they participated in the study. The study was conducted in accordance with the Declaration of Helsinki and the protocol was approved by the Human Ethics Committee of University of the Sunshine Coast (Project identification code A181095).

### 2.4. Data Collection

Structured interviews were guided by an interviewer-administered questionnaire divided into four sections; (1) participant characteristics (eight questions), (2) interest (two questions), (3) obstacles and enablers (five questions), and (4) expected costs and benefits (four questions) which is available from authors on request. Development of interview questions was based on relevant literature to answer the objectives of the study and were designed to determine participant’s social, economic, and nutritional wellbeing, and explore barriers and enablers to participating in seaweed harvesting, marketing, and consumption. Interview questions were translated into i-Kiribati and reviewed by a local researcher to check for ambiguity, appropriateness of wording, and cultural acceptability. The interview tool was piloted with eight South Tarawa residents, reviewed, and minor changes made to question order and wording to ensure questions were translated accurately and interpreted appropriated by participants.

Enumerators were selected by the Principal Fisheries Officer (Coastal Fisheries) from staff at the Ministry of Fisheries Apia, Samoa and Marine Resource Development Training Unit Tarawa, Kiribati. Four enumerators were trained in 2018 and a separate 4 enumerators were trained for the 2019 workshop. All enumerators were fluent in English and I-Kiribati. Training was conducted over two days and covered the questionnaire tool, ethical behaviour, and best practice for consistent data collection.

Each interview lasted from 30–40 min and took place in the community at the site of the training workshops. Enumerators documented all responses directly on the structured interview tool during the interview. After each open-ended question, the enumerator recounted a summary of the participant’s response as a means of respondent verification. At this time, participants were invited to provide feedback, clarification, and offer changes where needed to ensure enumerators had accurately captured participant responses.

### 2.5. Analysis

Quantitative data was entered into IBM Statistical Package for Social Sciences (SPSS version 24, SPSS Inc., Chicago, IL, USA, 2016). Descriptive analysis included frequencies, percentages, means, and standard deviations. Conventional content analysis of responses to open-ended questions, whereby categories and names for categories emerged from the data, was conducted to uncover common themes. To increase the trustworthiness of qualitative analysis, two members of the research team independently coded all responses before coming together to agree upon common themes and resolve discrepancies through discussion.

## 3. Results

### 3.1. Demographic Characteristics

A total of 49 participants, representing 13 villages in Tarawa, took part in the workshops (workshop 1 *n* = 24 and workshop 2 *n* = 25) and all participants were interviewed and included in this study (Table 2). Participants were women aged between 24 and 71 years, and a high proportion (82%) were married. Household size ranged from one to 19 people living in the home. Main sources of household income were government jobs (37%), fishing (14%), remittance (10%), and small business (41%) which included running a small shop selling cigarettes, vegetables, or hand sewn products.

### 3.2. Interest and Motivations for Involvement in the Seaweed Food Supply Chain

**Figure 2:**
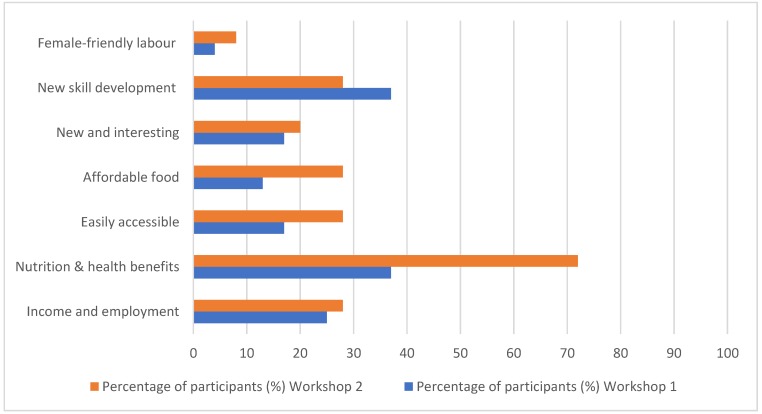
Identified motivators for involvement in seaweed harvesting, marketing, and consumption from Workshop 1 (24 participants in 2018) and Workshop 2 (25 participants in 2019).

All women felt that if promoted, the people of Kiribati would be interested in eating seaweed. As part of the first workshop, participants agreed upon a local name for *Caulerpa*, referring to them in i-Kiribati as *kureben taari* (“grapes from the sea”). Content analysis of open-ended responses revealed several key motivators for involvement across the supply chain (Figure 2).

Women noted that watching the Samoan seaweed harvesters in the field filled them with confidence to collect sea grapes from the reef at low tide. This common theme is illustrated through the following quote:

“The training program was useful when we were able to go to the field and take part with the practical exercise. Watching the Samoan women allowed me to learn and remember the know-how. They show me it is so easy.”

*[Workshop 1 Participant 7, age 35]*

**Figure 3:**
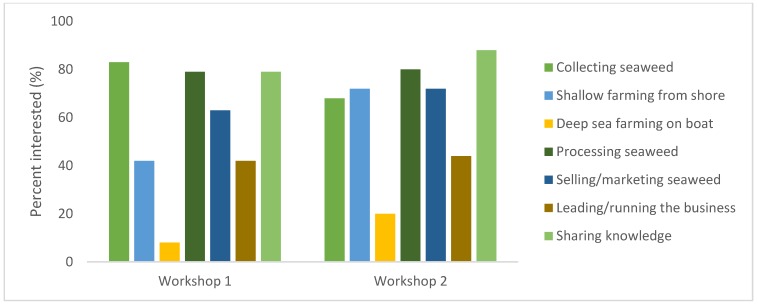
Participant interest in involvement across the seaweed food chain. Workshop 1 (24 participants in 2018) and Workshop 2 (25 participants in 2019).

There was high interest in being involved in most activities across the seaweed food supply chain (Figure 3). Almost all women in each workshop (92%) felt they had time to dedicate to seaweed harvesting, processing, and/or marketing. The amount of time women felt they could contribute ranged from two hours per week to three days per week. Many women specified certain days that seaweed work would fit in their own and their friend’s weekly schedule. A short film on the peer-to-peer training was made during the first workshop in 2018 and is available for additional context of group activities and insights from participants (https://www.youtube.com/watch?v=JhysbGxHfcI&t=5s).

Common enablers to ongoing participation in seaweed harvesting, processing, and consumption included, further training, social support, equipment (including safety boots to walk on the reef), recipes, and promotion and awareness raising in the community. These themes are illustrated through the following quotes.

*“I request some training and skills on how to market seaweed, what business things need to be planned for before marketing.”*

*[Workshop 1 Participant 2; age 39]*

*“If I can go to collect seaweed with someone, I will feel safer, so maybe I will go with my niece or neighbour.”*

*[Workshop 1 Participant 21; age 54]*

*“We need awareness and promotion on the radio on the importance of eating sea grapes for your health.”*

*[Workshop 2 Participant 8; age 39]*

### 3.3. Barriers and Obstacles to Involvement in the Seaweed Food Supply Chain

**Figure 4:**
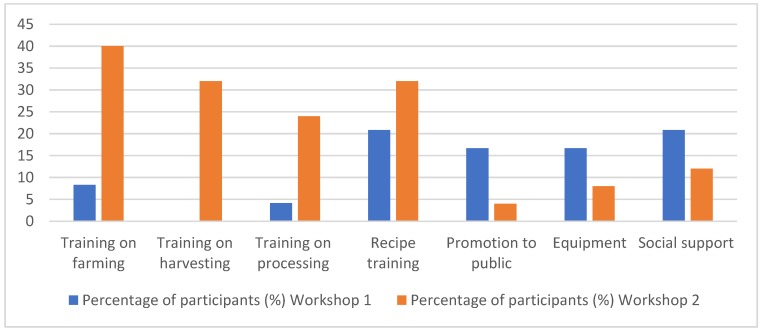
Factors identified by women to support them in ongoing engagement across the seaweed Figure 1. (24 participants in 2018) and Workshop 2 (25 participants in 2019).

Women attending both workshops identified barriers to ongoing engagement across the seaweed food supply chain, these included further training, promotion and public awareness, and social support. Those attending the first training workshop (focused on seaweed harvesting and processing) requested further support with recipe development and cooking skills, whereas women attending the second workshop (seaweed recipes and nutrition) felt they needed more training in seaweed farming, harvesting, and processing (Figure 4).

### 3.4. Expectations and Benefits From Involvement in the Seaweed Food Supply Chain

**Figure 5:**
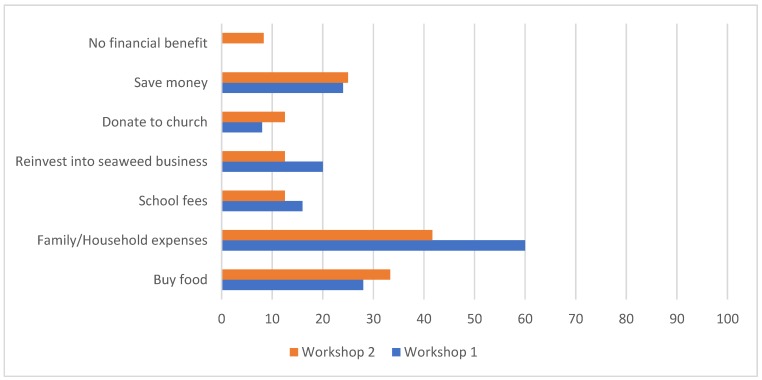
Expectations for financial benefits from money earned through involvement in seaweed harvesting and marketing. Workshop 1 (24 participants in 2018) and Workshop 2 (25 participants in 2019).

All women recognised the income generating opportunity that selling seaweed offers. The recommended asking price varied between AU $1 and AU $5 per bundle containing around 500 g fresh weight (median stated price was AU $2 with 50% of women stating between AU $2 and AU $3 as an acceptable price they would pay per bundle; note Australian dollar, AUD, is the currency of Kiribati). Approximately one quarter of women in each workshop (25% in 2018 and 28% in 2019) were not involved in decisions relating to how money was spent in the household. In these cases, financial decisions were made by the woman’s son or husband. For those women who made financial decisions themselves or in collaboration with other household members, they intended to use financial benefits from seaweed harvesting and marketing to contribute to family and household expenses (such as bus fare and soap), buying food from the store, towards personal savings, children’s education, and church donations (Figure 5). Two women participating in the 2019 nutrition and recipe workshop did not identify any financial benefits from involvement in the seaweed food chain.

Women were eager to share their learning from attending training workshops with their friends and family. In addition to income generating opportunities, other personal and social benefits that women identified included improved community cohesion, as well as access to a healthy and nutritious food source. These common themes are illustrated through the following quotes.

*“I will be popular, and when I share my new skills it will join the community together, some neighbours are shy so we can include them to join in. And it will mean food for health, for all.”*

*[Workshop 1 Participant 4, age 45]*

*“We get what our body needs from the seaweed, all the health and nutrition. Vegetables from the store are very expensive, so collecting seaweed will save us money.”*

*[Workshop 1, Participant 24, age 45]*

## 4. Discussion

This study aimed to determine interest and explore barriers and enablers for i-Kiribati women to participate in developing a seaweed-based food supply chain using existing edible seaweed resources available on the atolls. Given more than half of household expenditure goes towards imported foods [25], the high prevalence of multiple micronutrient deficiencies, and poor dietary diversity seen in this population (10% of people eating vegetables and 5% eating fruit) [10], understanding how to increase sustainable food production to create a healthier food environment is important to address diet-related chronic disease alongside broader ecological and socioeconomic dimensions of food choice. Our study focused on women given their role in i-Kiribati culture is predominantly in food procurement and preparation, as well as the significant role women play in seaweed harvesting and processing in other countries across the Indo-Pacific [26,27,28].

Seaweed farming is low cost, requires minimal investment, can be operated at a family level, and is compatible with traditional farming methods, all of which can reduce the environmental impact of land-based agriculture [15]. Seaweeds grow abundantly in oceans under various climatic conditions, they contain many essential nutrients, they do not require exogenous feeding for their cultivation, and have minimal impact on the environment, thus making them increasingly recognised as a sustainable food source with the potential to play a major role in providing food security worldwide [29]. Of the three species of seaweed evaluated in this study, one is farmed (*Kappaphycus alvarezii*) but is presently produced only for export into the carrageenan gel market and is not used in Kiribati as a food. The edible *Caulerpa* sea grapes, are found on the reef flat on the ocean side of the island, was familiar to some participants but not widely utilised, whereas *Acanthophora* is an agar gel producing seaweed that occasionally blooms on the reef flat and washes up as a beach-wrack. Other edible Pacific Island seaweed varieties (for example, different species of *Caulerpa*, *Gracilaria*, *Hypnea*, *Halymenia*, and *Ulva*) provide a strong and complementary nutritional base for improving health outcomes based on their unique biochemical profiles [20]. There was a high level of intertest from women in our study to be involved in most activities across the seaweed food chain. Women were motivated by the ease of harvesting seaweed for food as well as the nutritional benefits associated with eating seaweed. Poor access and availability to fresh nutritious foods, alongside a lack of skills and motivation, are key determinants of poor health outcomes [14]. Providing women with the skills and confidence to source and prepare local seaweeds, as done in this study, may increase food literacy around procurement and preparation of edible seaweeds, reducing food expenses, and potentially improving diet quality.

The move to urbanisation has reduced the knowledge of old farming or fisheries techniques [30,31]. This shortage of skills and knowledge is a potential barrier for improving seaweed wild-harvest and farming, and in turn its consumption [32]. Education and training support is thus essential to overcome these barriers, whilst also raising community awareness of the potential socio-economic benefits of seaweed farming, enticing more individuals to get involved [15]. Our study took a peer-led approach to training, using Samoan seaweed farmers and Fisheries officers through a process of peer education, which has been shown to improve the efficiency and effectiveness of community-based interventions [33]. Peer-led strategies have been used in a variety of settings to effectively target a broad range of health outcomes such as smoking cessation, asthma management, mental health, and addiction [34], as well as to improve diet and nutrition behaviour [35,36,37]. The cross-country approach taken in our study may be the foundation of interdisciplinary research and collaboration between Kiribati and Samoa, where fisheries officers have greater confidence to think outside the box in terms of new opportunities that sit at the intersection of fisheries and sustainable development. Despite women’s enthusiasm to share their new knowledge and skills within their own village, this did not appear to happen between the first and second workshops. We need to develop strategies and systems that better support women to become peer trainers to ensure new learnings are disseminated broadly. We see the opportunity to continue with more peer-to-peer learning for (1) educating other people on Tarawa and the outer islands (as women felt enabled and excited to share their new knowledge and skills) and (2) more advanced peer-to-peer training of supply chain/marketing/scale from other countries (for example, broadening the scope through other edible seaweeds used in Fiji and Tonga) [38,39].

Social sustainability is central to the ability for a local food system to deliver nutritious diets to the community. Social vulnerability was evident in our sample of women. Women in our study were more interested in collecting seaweed from the reef at low tide, where they felt safer, as opposed to shallow water farming or deep-sea farming from a boat. Social support was identified by women in both workshops as a factor to enhance involvement in activities across the supply chain. Social sustainability considers how the world produces and consumes foods and is central to achieving Sustainable Development Goal 2 (SDG2), ensuring food and nutrition security within a sustainable food system [40]. The targets of SDG2 also include goals that relate to gender equality, health, and poverty, all connected through the multiple inputs, activities, and actors that comprise the food system [41]. To achieve SDG2, all actors must work together to form socially sustainable solutions. The peer-led approach taken in this study provided the social support that our women desired, in the short term, however, once training ceased and peer-trainers departed, the desire for social support will likely remain. Further research is warranted to identify ways to build social sustainability across the seaweed food supply chain, for example, approaches to community connectedness, mentoring, and buddy systems.

Although income generation was a key motivator for many women, we found that approximately one quarter of women in each workshop (25% in Workshop 1 and 28% in Workshop 2) were not involved in decisions relating to how money was spent in the household. Our findings highlight the need to increase the capacity of some women to participate in financial (and other) decision making. Empowerment is, among other aspects, about changing power relations in order to enhance the ability of vulnerable groups to shape their lives and to improve the terms of their participation in the value chain [42,43]. To ensure social sustainability, we must consider fairness and not show favouritism among actors along the food chain [19]. Thus, engaging whole communities, both men and women, from all socioeconomic backgrounds, is needed to empower women and their families to establish new seaweed supply chains that are sustainable in all aspects of society.

Kiribati has the highest household spending on imported foods of all Pacific Island nations, making up 53% of their total household food expenditure [25]. This is particularly concerning given the World Development Indicators rank Kiribati as the poorest country in the Indo-Pacific with a gross domestic product (GDP) per capita of $2.290 [44]. Income generation was a key motivator for women in this study to be involved in seaweed work. The move from copra to seaweed (*Kappaphycus alvarezii)* farming as an export commodity has previously been a successful transition in Tabuaeran (Fanning Island), part of the Line Islands of Kiribati [20]. In this instance, copra was the only source of income in the Line Islands, which was limited by low-yielding senile trees and coconut rat infestation. The move to seaweed farming substantially improved well-being and increased economic growth for this population [20]. Women in our study were motivated by the prospect of earning money, which is encouraging as we know that engagement across the seaweed food chain can reduce poverty by creating income and employment, especially in rural areas that lack other income-generating opportunities [15]. In addition to earning money, the inclusion of women workers in the seaweed food supply chain may mitigate aspects of traditional society where women take on a homemaker role, which work against the progress of women, thus benefiting the whole family and community [20].

## 5. Conclusions

Our study provides insight into how to support women’s engagement in developing new seaweed-based food supply chains. The qualitative approach taken in this study enabled us to understand experiences of a group of people whose voice is not often heard. Although not generalisable to the broader population, we have uncovered strategies to support women’s engagement in an industry that offers potential economic, social, and nutritional benefits. The experiences of our participants are likely not unique given the common domestic role that women play in households in Pacific Island countries. Recognising women’s roles and motives in food supply chains, and addressing their barriers and enablers to participation, are central to developing sustainable food systems and achieving food and nutrition security. This study confirmed women’s enthusiasm for involvement in seaweed harvesting, marketing, and consumption and offers insight into how peer-led seaweed training workshops can assist towards improved social, economic, and nutritional wellbeing. Further participatory research to gain in-depth understanding of how to accommodate women’s as well as men’s diverse needs and address issues that traverse the supply chain is warranted. Gender inclusive activities are needed to explore and build roles that are sustainable for women and their families. Such understanding can inform the development of targeted support and engagement of women in creating new short supply chains to generate income, encourage sustainable diets, and promote personal and community wellbeing.

## Figures and Tables

**Table 1:** Species of seaweed utilised in training workshops on the food supply chain.

Seaweed	*Caulerpa*	*Kappaphycus*	*Acanthophora*
**Taxonomy**	Green seaweed, *C. racemosa* and *C. chemnitzia*	Red seaweed, *K. alvarezii*	Red seaweed, *A. spicifera*
**Source**	Reef flat and crest, South Tarawa	Farmed, Tabuaeran (Fanning Island)	Reef flat, South Tarawa
**Key features**	Sea grapes, consumed fresh	Carrageenan gel, thickener	Agar gel, thickener
**Seaweed form**	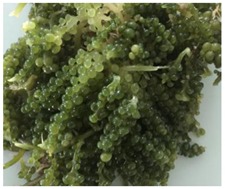	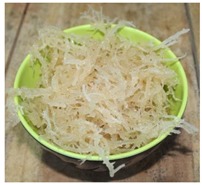	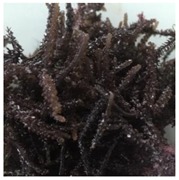
**Product example**	Sea grape salad 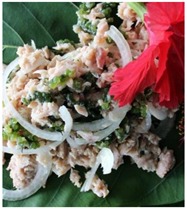	*Kappaphycus* tuna balls 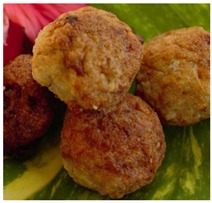	*Acanthophora* papaya jam 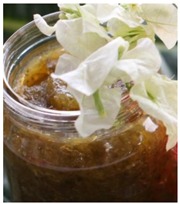

**Table 2:** Descriptive characteristics of participants attending the seaweed training workshops.

Characteristics	Workshop 1 (*n* = 24)	Workshop 2 (*n* = 25)
**Age mean (± SD)**	47 (±11.41)	38 (±10.44)
**Gender *n* (%)**	-	-
**Male**	0 (0)	0(0)
**Female**	24 (100)	25 (100)
**Marriage *n* (%)**	-	-
**Yes**	18 (75)	22 (88)
**No**	0 (0)	2 (8)
**Not anymore**	6 (25)	1 (4)
**Children mean (±SD)**	3.75 (±2.11)	3.17 (±1.69)
**Household size mean (±SD)**	6.42 (±4.11)	9.72 (±6.96)
**Source of income *n* (%)**	-	-
**Fishing**	2 (8)	5 (20)
**Business**	14 (58)	6 (24)
**Government**	6 (25)	12 (48)
**Remittance**	3 (12.5)	2 (8)
**Other**	12 (50)	17 (68)
**Attended 2018 workshop *n* (%)**	24 (100)	6 (24)
